# Effect of the Combination of Concomitant Drugs on Efficacy of Immune Checkpoint Inhibitors in Non‐Small Cell Lung Cancer

**DOI:** 10.1002/cnr2.70399

**Published:** 2025-11-06

**Authors:** Masafumi Saiki, Kazuho Takusagawa, Nozomu Takahashi, Kenta Homma, Tsukasa Satoh, Satoshi Furuya, So Shimamura, Chisa Omori, Hiroki Ohkoshi, Yuki Hoshino, Yoshinori Uchida, Shinnosuke Ikemura, Kenzo Soejima

**Affiliations:** ^1^ Department of Respiratory Medicine, Graduate School of Medicine University of Yamanashi Chuo Yamanashi Japan

**Keywords:** concomitant drugs, immune checkpoint inhibitor, non‐small cell lung cancer, proton pump inhibitor

## Abstract

**Background:**

Emerging evidence indicates that baseline use of certain concomitant drugs may affect the efficacy of immune checkpoint inhibitors (ICIs), including PD‐1, PD‐L1, and CTLA‐4 inhibitors, in patients with cancer. However, most previous studies have evaluated individual drug classes in isolation, without considering potential interactions among multiple drugs.

**Aims:**

This study aimed to evaluate the individual and combined effects of commonly prescribed concomitant drugs on the efficacy and safety of ICI‐based therapy in patients with non‐small cell lung cancer (NSCLC).

**Methods:**

We conducted a retrospective analysis of 124 patients with advanced or recurrent NSCLC who received first‐line ICI‐based treatments at a single institution. Drug exposure at treatment initiation was assessed for proton pump inhibitors (PPIs), low‐dose aspirin, non‐steroidal anti‐inflammatory drugs, statins, biguanides, antibiotics, and probiotics. Associations with progression‐free survival (PFS), overall survival (OS), and immune‐related adverse events (irAEs) were analyzed using multivariate Cox regression models.

**Results:**

PPI use was independently associated with shorter PFS (HR: 2.44, *p* < 0.001) and OS (HR: 2.04, *p* = 0.01). In contrast, low‐dose aspirin use was independently associated with longer PFS (HR: 0.31, *p* = 0.01). Patients receiving both PPIs and aspirin had longer PFS and OS compared to those receiving PPIs alone, although the differences were not statistically significant. No consistent associations were observed for other drugs. The incidence of irAEs was not significantly affected by concomitant drug use.

**Conclusion:**

PPI use at baseline may be associated with reduced efficacy of ICI therapy in NSCLC patients. In contrast, low‐dose aspirin use was independently associated with improved PFS, and may potentially mitigate the negative effects of PPIs. These findings underscore the importance of considering concomitant drug use when initiating ICI treatment. Prospective studies are needed to validate these observations and clarify underlying mechanisms.

## 1 Introduction

Non‐small cell lung cancer (NSCLC) accounts for approximately 85% of all lung cancer cases and remains a leading cause of cancer‐related mortality worldwide, particularly in its advanced stages despite recent therapeutic advancements [1]. Immune checkpoint inhibitors (ICIs), particularly PD‐1/PD‐L1 inhibitors such as nivolumab [2, 3], pembrolizumab [4], and atezolizumab [5], have revolutionized NSCLC treatment by significantly prolonging survival. These agents, alone or in combination with cytotoxic chemotherapy or CTLA‐4 inhibitors, have become the standard of care in both first‐ and second‐line settings [6, 7, 8, 9, 10].

However, despite the clinical success of ICI therapy, a substantial proportion of patients do not derive long‐term benefit. This has led to extensive efforts to identify predictive biomarkers of ICI efficacy, such as PD‐L1 expression and tumor mutational burden. More recently, emerging research has suggested that the baseline use of certain concomitant drugs including antibiotics, proton pump inhibitors (PPIs), and aspirin may influence ICI outcomes, likely through immunomodulatory effects or alterations in the gut microbiome [11, 12]. However, most previous studies have evaluated these drugs individually, often focusing on a single drug class without accounting for potential interactions among multiple agents. Given that polypharmacy is increasingly common among patients with cancer, especially older individuals, and the potential for drug–drug interactions affecting immune function is substantial [13]. Furthermore, the clinical indications for these drugs—for example, aspirin for old myocardial infarction or PPIs for non‐steroidal anti‐inflammatory drugs (NSAIDs)–induced gastrointestinal protection—may themselves reflect underlying disease severity, leading to confounding.

To address these gaps, the present study aimed to evaluate both the individual and combined effects of several commonly prescribed concomitant drugs on the efficacy and safety of ICI‐based therapy in patients with NSCLC. Particular attention was given to PPI, which has been associated with disruption of the gut microbiota, and to low‐dose aspirin, which may exert immunomodulatory effects through antiplatelet activity. By analyzing these drugs within a single, integrated framework, this study seeks to provide a more comprehensive understanding of how polypharmacy may influence clinical outcomes in patients undergoing ICI therapy.

## 2 Methods

### 2.1 Study Design and Patient Selection

This retrospective cohort study was conducted at the Department of Respiratory Medicine, University of Yamanashi Hospital, by reviewing medical records of patients diagnosed with NSCLC between April 2018 and March 2024. Eligible patients met the following criteria: histologically or cytologically confirmed NSCLC; stage III disease not eligible for definitive chemoradiotherapy, stage IV disease, or recurrence after curative surgery; received ICI‐based therapy as first‐line systemic treatment. Exclusion criteria included: diagnosis of large cell neuroendocrine carcinoma, incomplete data on concomitant drug use at treatment initiation, baseline systemic corticosteroids use for autoimmune disease or brain metastasis at treatment initiation. Baseline demographic and clinical information was extracted from electronic health records, including age, sex, smoking history, Eastern Cooperative Oncology Group performance status (ECOG PS), tumor histology, clinical stage, PD‐L1 tumor proportion score, ICI regimen (monotherapy or combination with chemotherapy and/or CTLA‐4 inhibitors), treatment response, progression‐free survival (PFS), overall survival (OS), and immune‐related adverse events (irAEs). Given the heterogeneity of ICI regimens, the use of cytotoxic chemotherapy was included as a covariate in the multivariate analysis to adjust for potential confounding effects on survival outcomes.

### 2.2 Assessment of Concomitant Drugs

We assessed concomitant drug use at the time of first‐line ICI treatment initiation. The following drug classes were included, based on previous literature implicating potential immunomodulatory or microbiome‐altering effects: PPIs; low‐dose aspirin (≤ 100 mg/day); NSAIDs; statins; biguanides; probiotics; and antibiotics. Drug exposure was defined as follows: for all drugs except antibiotics, exposure was defined as active prescription at the time of ICI initiation. For antibiotics, exposure was defined as any systemic antibiotic administration within 30 days prior to ICI initiation. Due to the retrospective nature of the study, detailed information on the duration, type, or clinical indication of antibiotic use was not uniformly available and thus could not be included in the analysis. Each drug was recorded as a binary variable (yes/no), and patients were classified as “exposed” if they met the criteria above.

### 2.3 Outcomes and Definitions

The primary outcomes were PFS and OS. PFS was defined as the time from ICI initiation to documented disease progression or death from any cause. OS was defined as the time from ICI initiation to death from any cause. The secondary outcome was the objective response rate (ORR) and the incidence and spectrum of irAEs, which were identified based on physician documentation and graded using the Common Terminology Criteria for Adverse Events (CTCAE) version 5.0.

### 2.4 Statistical Analysis

All experimental data are presented as median and range. Baseline characteristics were compared using Fisher's exact test for categorical variables and the Mann–Whitney *U*‐test for continuous or ordinal variables. PFS and OS were calculated using the Kaplan–Meier method and compared using the log‐rank test. Multivariate Cox proportional hazards regression models were constructed separately for PFS and OS to identify factors independently associated with each outcome. Variables considered for inclusion were selected based on clinical relevance and prior literature, including age, sex, ECOG performance status, PD‐L1 expression, smoking history, cytotoxic chemotherapy use, and concomitant drug exposure. Due to the relatively small sample size and to avoid model overfitting, variable selection for each model was performed using the Akaike Information Criterion (AIC) with a stepwise approach. Variables with low prevalence, such as biguanides and probiotics, were excluded from candidate variables prior to selection. PD‐L1 status was unavailable for 5.6% of patients. For multivariate Cox regression analyses, these cases were excluded to avoid introducing an undefined category into the model. However, for descriptive statistics and unadjusted Kaplan–Meier analyses, these patients were included to preserve the overall cohort size and reflect real‐world clinical data completeness. We also conducted interaction analyses to assess whether the association between PPI use and survival outcomes varied by age (≥ 75 vs. < 75) or performance status (0–1 vs. 2–4). Interaction terms were included in the Cox proportional hazards models to evaluate potential effect modification. A *p*‐value of less than 0.05 was considered statistically significant. All statistical analyses were performed using EZR (Saitama Medical Center, Jichi Medical University), a graphical user interface for R (The R Foundation for Statistical Computing) [14]. EZR is a modified version of the R Commander designed to include statistical functions commonly used in biostatistics.

### 2.5 Ethics Approval

This study was conducted in accordance with the ethical standards of the Declaration of Helsinki and was approved by the Ethics Committee of the University of Yamanashi (approval number: 2823). Due to the retrospective nature of the study, the requirement for written informed consent was waived.

## 3 Results

### 3.1 Patient Characteristics

A total of 184 patients diagnosed with NSCLC were initially assessed for eligibility. Sixty patients were excluded for the following reasons: 47 did not receive first‐line treatment, four were diagnosed with large cell neuroendocrine carcinoma (LCNEC), and nine were receiving systemic corticosteroids. The remaining 124 patients met the inclusion criteria and were included in the final analysis (Figure S1). Baseline characteristics are summarized in Table 1. The median age was 71.5 years (range, 34–93), with 45 patients (36.3%) aged over 75 years. ECOG PS was 0–1 in 96 patients (77.4%) and 2–4 in 28 patients (22.6%). Histologically, 68 patients (54.8%) had adenocarcinoma, 35 patients (28.3%) had squamous cell carcinoma, and 21 patients (16.9%) had other subtypes. PD‐L1 tumor proportion scores were < 1% in 40 patients (32.3%), 1%–49% in 41 patients (33.1%), and ≥ 50% in 36 patients (29.0%). Treatment regimens included chemotherapy plus a PD‐1/PD‐L1 inhibitor in 74 patients (59.7%), chemotherapy plus a PD‐1 inhibitor and a CTLA‐4 inhibitor in 15 patients (12.1%), a PD‐1/PD‐L1 inhibitor alone in 26 patients (21.0%), and a PD‐1 inhibitor plus a CTLA‐4 inhibitor in nine patients (7.3%).

**TABLE 1:** Baseline characteristics of patients.

		*n* = 124
Age	Median [range]	71.5 [34–93]
	< 74	79 (63.7)
	≤ 75	45 (36.3)
Gender (%)	Female	32 (25.8)
	Male	92 (74.2)
Smoking history (%)	Current/Former	104 (84.6)
	Never	19 (15.4)
ECOG PS (%)	0–1	96 (77.4)
	2–4	28 (22.6)
Histology (%)	Adenocarcinoma	68 (54.8)
	Squamous cell carcinoma	35 (28.3)
	Other	21 (16.9)
Stage (%)	III	6 (4.5)
	IV	91 (68.4)
	Recurrence	36 (27.1)
PD‐L1 (%)	< 1%	40 (32.3)
	1%–49%	41 (33.1)
	≤ 50%	36 (29.0)
	NA	7 (5.6)
Regimen (%)	Chemotherapy + PD‐1/PD‐L1	74 (59.7)
	Chemotherapy + PD‐1 + CTLA‐4	15 (12.1)
	PD‐1/PD‐L1	26 (21.0)
	PD‐1 + CTLA‐4	9 (7.3)

Abbreviations: Chemo, chemotherapy; CTLA‐4, cytotoxic T‐lymphocyte antigen‐4; ECOG PS, Eastern Cooperative Oncology Group performance status; PD‐(L) 1, programmed cell death (ligand) 1.

Concomitant drug use at the initiation of first‐line ICI therapy is summarized in Table 2. Among the 124 patients, the most commonly used drugs were as follows: PPIs, 46.0%; low‐dose aspirin, 12.9%; NSAIDs, 37.1%; statins, 26.6%; biguanides, 6.5%; and probiotics, 4.9%. Additionally, 37.9% of patients had received antibiotics within 30 days prior to ICI initiation.

**TABLE 2:** Concomitant drugs use.

Concomitant drug	*n* = 124
PPIs	57 (46.0)
Aspirin	16 (12.9)
NSAIDs	46 (37.1)
Statin	33 (26.6)
Biguanide	8 (6.5)
Antibiotics	47 (37.9)
Probiotics	6 (4.9)
PPIs + aspirin	12
PPIs + NSAIDs	28
PPIs + statin	19
PPIs + biguanide	6
PPIs + antibiotics	24
PPIs + probiotics	5

Abbreviations: NSAIDs, non‐steroidal anti‐inflammatory drugs; PPIs, proton pump inhibitors.

### 3.2 Incidence of irAEs

Details of the irAEs are summarized in Table S1. Among the 124 patients, 64 (51.6%) experienced a total of 84 irAEs. The incidence of irAEs was compared between patients who did and did not use each concomitant drug, as summarized in Table 3. However, no significant differences in the incidence of irAEs were observed for any of the evaluated drugs.

**TABLE 3:** Incidence of irAE.

Concomitant drugs		irAE (%)	*p*
Overall		51.6	
PPIs	Yes	54.4	0.593
	No	49.3	
Low‐dose aspirin	Yes	50.0	1
	No	51.9	
NSAIDs	Yes	45.7	0.355
	No	55.1	
Statin	Yes	42.4	0.230
	No	54.9	
Biguanide	Yes	75.0	0.275
	No	50.0	
Antibiotics	Yes	59.6	0.197
	No	46.8	
Probiotics	Yes	83.3	0.211
	No	50.9	

Abbreviations: NSAIDs, non‐steroidal anti‐inflammatory drugs; PPIs, proton pump inhibitors.

### 3.3 Therapeutic Effects

#### 3.3.1 Association Between the Outcome of ICI and Concomitant Drugs

The ORR was compared between patients with and without the use of each concomitant drug, as summarized in Table 4. The ORR was 41.5% in the PPI group and 63.5% in the non‐PPI group, with the ORR being significantly higher in the non‐PPI group. No significant differences were observed with any other concomitant drugs.

**TABLE 4:** Comparison of treatment outcomes between patients with and without concomitant drugs.

Concomitant drugs		ORR (%)	*p*
Overall		53.2	
PPIs	Yes	41.5	0.025
	No	63.5	
Low‐dose aspirin	Yes	37.5	0.188
	No	56.0	
NSAIDs	Yes	52.4	1
	No	54.1	
Statin	Yes	41.9	0.147
	No	57.6	
Biguanide	Yes	57.1	1
	No	53.2	
Antibiotics	Yes	55.6	0.849
	No	52.1	
Probiotics	Yes	60.0	1
	No	53.2	

Abbreviations: NSAIDs, nonsteroidal anti‐inflammatory drugs; PPIs, proton pump inhibitors.

**FIGURE 1:**
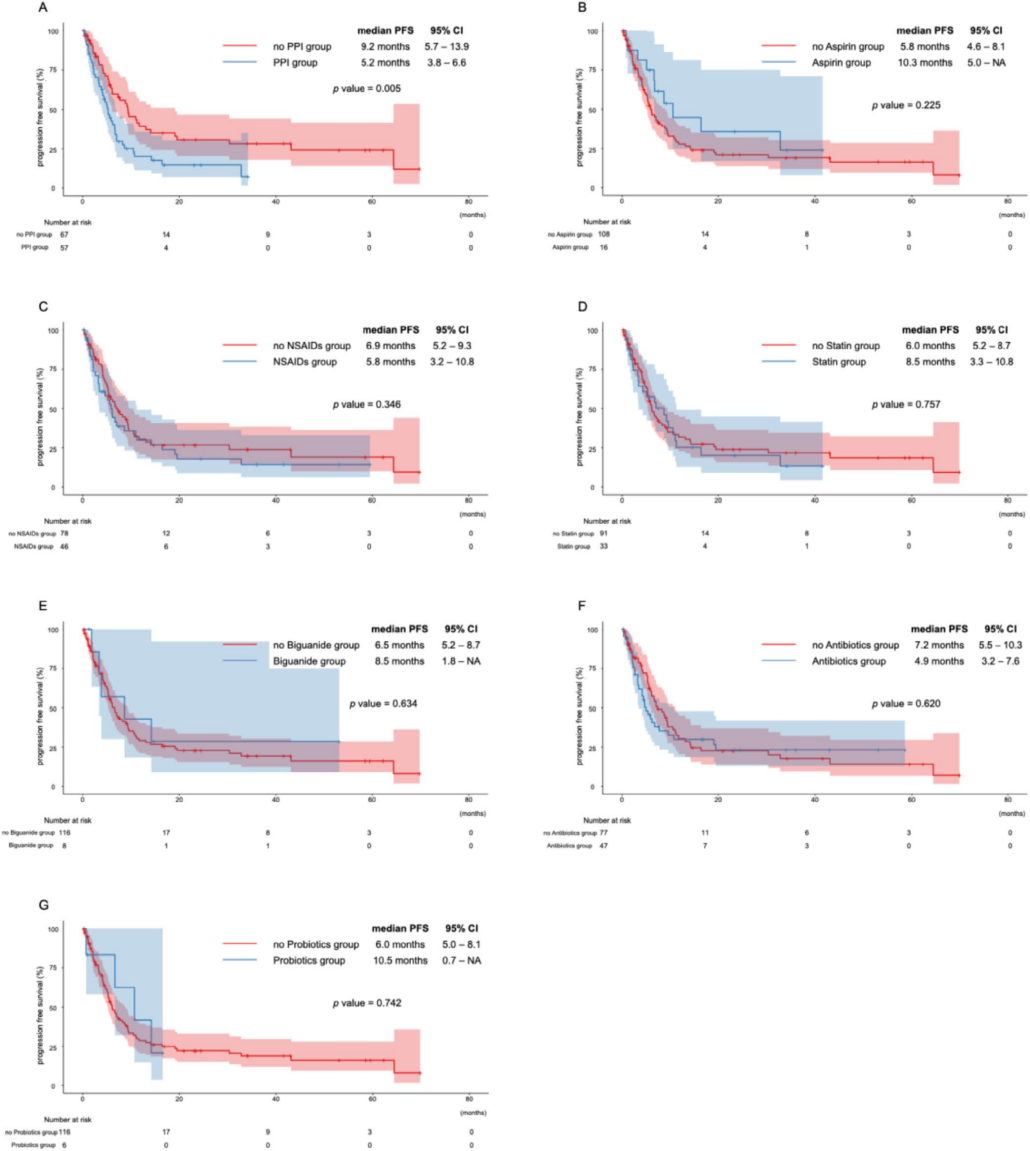
Progression‐free survival with or without each concomitant drug. (A) proton pump inhibitors, (B) low‐dose aspirin, (C) NSAIDs, (D) statins, (E) biguanides, (F) antibiotics, and (G) probiotics.

**FIGURE 2:**
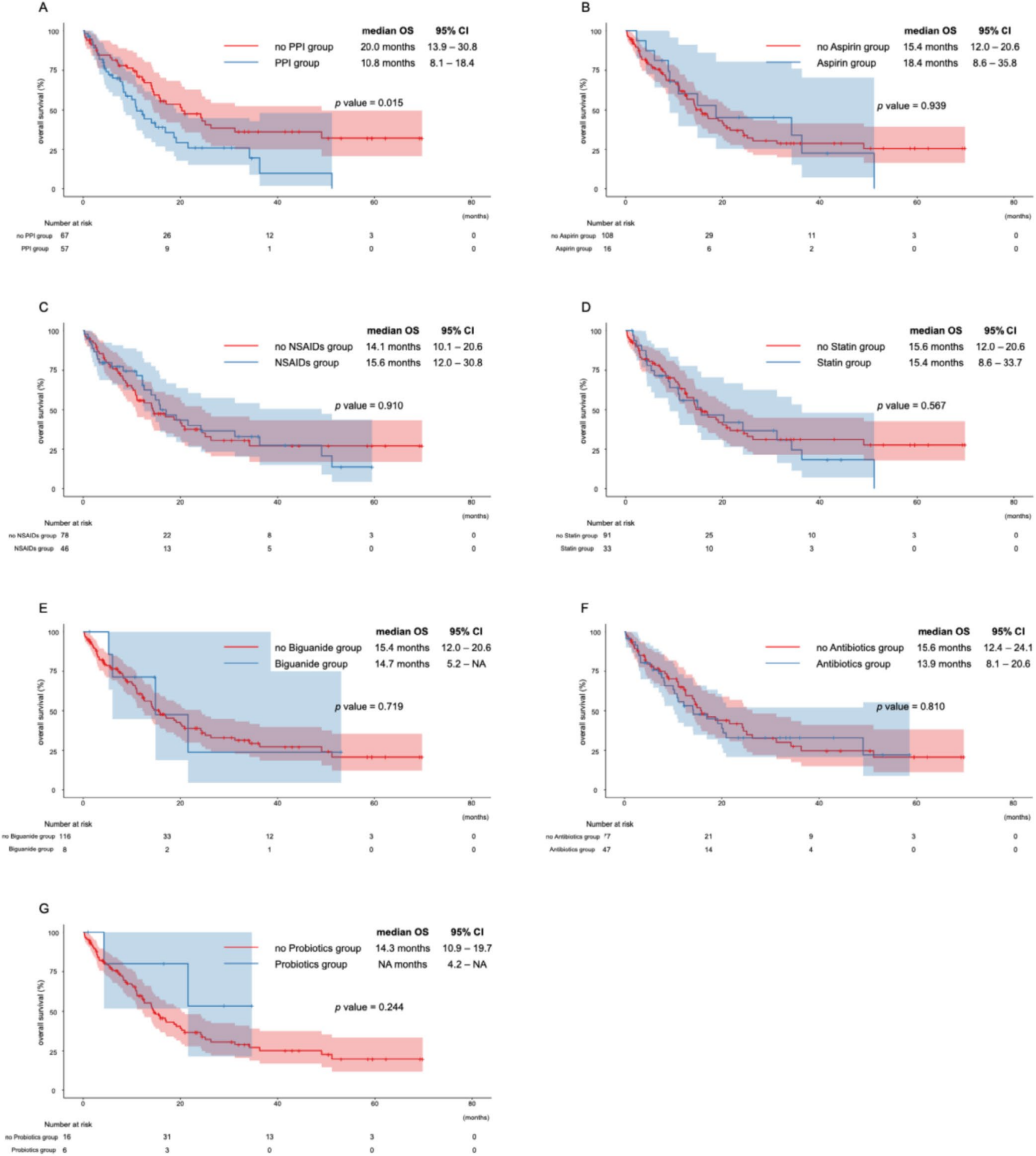
Overall survival with or without each concomitant drug. (A) proton pump inhibitors, (B) low‐dose aspirin, (C) NSAIDs, (D) statins, (E) biguanides, (F) antibiotics, and (G) probiotics.

PFS and OS were analyzed using Kaplan–Meier curves (Figures 1 and 2). The median PFS was 5.2 months (95% CI: 3.8–6.6) in the PPI group and 9.2 months (95% CI: 5.7–13.9) in the non‐PPI group. The median OS was 10.8 months (95% CI: 8.1–18.4) in the PPI group and 20.0 months (95% CI: 13.9–30.8) in the non‐PPI group. Significant differences were observed in PFS (*p* = 0.005), and OS (*p* = 0.015) between the two groups. The median PFS was 10.3 months (95% CI: 5.0–NA) in the low‐dose aspirin group and 5.8 months (95% CI: 4.6–8.1) in the non‐aspirin group. The median OS was 18.4 months (95% CI: 8.6–35.8) and 15.4 months (95% CI: 12.0–20.6), respectively. Although these differences were not statistically significant (PFS: *p* = 0.225, OS: *p* = 0.939), a favorable trend was observed in the low‐dose aspirin group. No significant differences in PFS or OS were observed with or without any other concomitant medicines.

To further assess whether the effects of concomitant PPI or low‐dose aspirin use on ICI efficacy differ by PD‐L1 expression status, we conducted stratified analyses of PFS and OS according to PD‐L1 tumor proportion score (< 1%, 1%–49%, and ≥ 50%) (Figures S2 and S3). Among patients with PD‐L1 < 1%, PPI use was associated with a numerically shorter PFS (median 4.9 vs. 7.6 months; *p* = 0.063) and OS (9.9 vs. 15.6 months; *p* = 0.252). In the PD‐L1 1%–49% subgroup, PPI use was significantly associated with shorter PFS (5.2 vs. 9.4 months; *p* = 0.048) and showed a non‐significant trend toward reduced OS (12.0 vs. 20.6 months; *p* = 0.091). In contrast, no meaningful difference in PFS or OS was observed between PPI users and non‐users in the PD‐L1 ≥ 50% subgroup. Regarding low‐dose aspirin, no statistically significant differences in PFS or OS were found in any PD‐L1 subgroup.

As this was an exploratory study, unadjusted Kaplan–Meier survival curves were used to illustrate overall trends. We acknowledge that these curves may be influenced by confounding factors and should be interpreted with caution.

#### 3.3.2 Factors Associated With Therapeutic Effects (Table 5)

**TABLE 5:** Multivariate analysis.

Full model	PFS		OS	
	HR (95% CI)	*p*	HR (95% CI)	*p*
Age (≤ 75)	1.09 (0.63–1.88)	0.75	1.25 (0.70–2.22)	0.44
Sex (Male)	1.05 (0.56–1.96)	0.88	1.45 (0.73–2.88)	0.29
ECOG PS 2–4 (vs. 0–1)	1.65 (0.92–2.97)	0.095	2.51 (1.39–4.54)	0.0024
Smoking history (never)	0.95 (0.43–2.13)	0.91	1.24 (0.53–2.90)	0.62
With chemotherapy (+)	1.64 (0.87–3.10)	0.12	1.02 (0.52–2.00)	0.96
PD‐L1				
1%–49% (vs. < 1%)	0.69 (0.38–1.23)	0.2	0.69 (0.36–1.31)	0.26
50% (vs. < 1%)	0.88 (0.46–1.65)	0.68	0.90 (0.44–1.81)	0.76
Concomitant drugs				
Aspirin	0.36 (0.16–0.84)	0.018	0.57 (0.23–1.42)	0.23
NSAIDs	1.06 (0.63–1.80)	0.82	0.96 (0.54–1.69)	0.88
PPI	2.12 (1.30–3.46)	0.0026	1.78 (1.04–3.04)	0.034
Statin	1.50 (0.80–2.82)	0.2	1.18 (0.57–2.44)	0.65
Antibiotics	0.98 (0.59–1.63)	0.94	1.02 (0.60–1.76)	0.93
AIC‐selected model				
ECOG PS 2–4 (vs. 0–1)	1.57 (0.92–2.67)	0.099	2.21 (1.32–3.72)	0.0027
With chemotherapy (+)	1.54 (0.91–2.62)	0.11	NS	—
Aspirin	0.38 (0.17–0.87)	0.021	NS	—
PPI	2.17 (1.34–3.51)	0.0016	1.72 (1.05–2.80)	0.031
Statin	1.58 (0.87–2.86)	0.13	NS	—

Abbreviations: ECOG PS, Eastern Cooperative Oncology Group performance status; NS, not selected; NSAIDs, non‐steroidal anti‐inflammatory drugs; PD‐L1, programmed cell death ligand 1; PPIs, proton pump inhibitors.

In the multivariate Cox proportional hazards analyses excluding variables with low prevalence such as biguanides and probiotics, PPI use was significantly associated with poorer PFS (HR: 2.12, 95% CI: 1.30–3.46, *p* = 0.003) and OS (HR: 1.78, 95% CI: 1.04–3.04, *p* = 0.034). Aspirin use was significantly associated with improved PFS (HR: 0.36, 95% CI: 0.16–0.84, *p* = 0.018), but not OS. ECOG PS of 2–4 was significantly associated with worse OS (HR: 2.51, 95% CI: 1.39–4.54, *p* = 0.002) and showed a trend toward worse PFS (HR: 1.65, 95% CI: 0.92–2.97, *p* = 0.095).

After variable selection using the AIC, PPI use (HR: 2.17, 95% CI: 1.34–3.51, *p* = 0.0016) and aspirin use (HR: 0.38, 95% CI: 0.17–0.87, *p* = 0.021) remained significant predictors for PFS. For OS, PS (HR: 2.21, 95% CI: 1.32–3.72, *p* = 0.0027) and PPI use (HR: 1.72, 95% CI: 1.05–2.80, *p* = 0.031) were retained as significant factors. Other variables such as age, sex, smoking status, PD‐L1 expression, chemotherapy regimen, NSAIDs, statins, and antibiotics were not significantly associated with survival outcomes in the multivariate models.

To further assess the robustness of our findings, we performed interaction analyses to examine whether the association between PPI use and survival outcomes varied by age (≥ 75 vs. < 75) or performance status (PS 0–1 vs. 2–4). The results showed no statistically significant interactions between PPI use and either age or PS for both PFS and OS (Table S2). These findings suggest that the impact of PPI use on survival outcomes did not differ significantly across age or PS subgroups, supporting the overall consistency and generalizability of the observed associations.

Although the model included the presence or absence of chemotherapy, further stratification by specific ICI regimens (e.g., monotherapy vs. combination with CTLA‐4 inhibitors) was not performed due to statistical limitations. This limitation is acknowledged and should be addressed in future studies.

### 3.4 PFS and OS in Patients Receiving Both PPI and Low‐Dose Aspirin or Other Concomitant Drugs

Given the significant associations observed in the multivariate analysis—where PPI use was linked to poorer outcomes and low‐dose aspirin use to improved PFS—we further explored the combined effects of these drugs on the efficacy of PD‐1/PD‐L1 inhibitor‐based therapy. At baseline, 12 patients were receiving both PPI and aspirin, 28 were receiving both PPI and NSAIDs, 19 were receiving both PPI and statins, six were receiving both PPI and biguanides, 24 were receiving both PPI and antibiotics, and five were receiving both PPI and probiotics.

**FIGURE 3:**
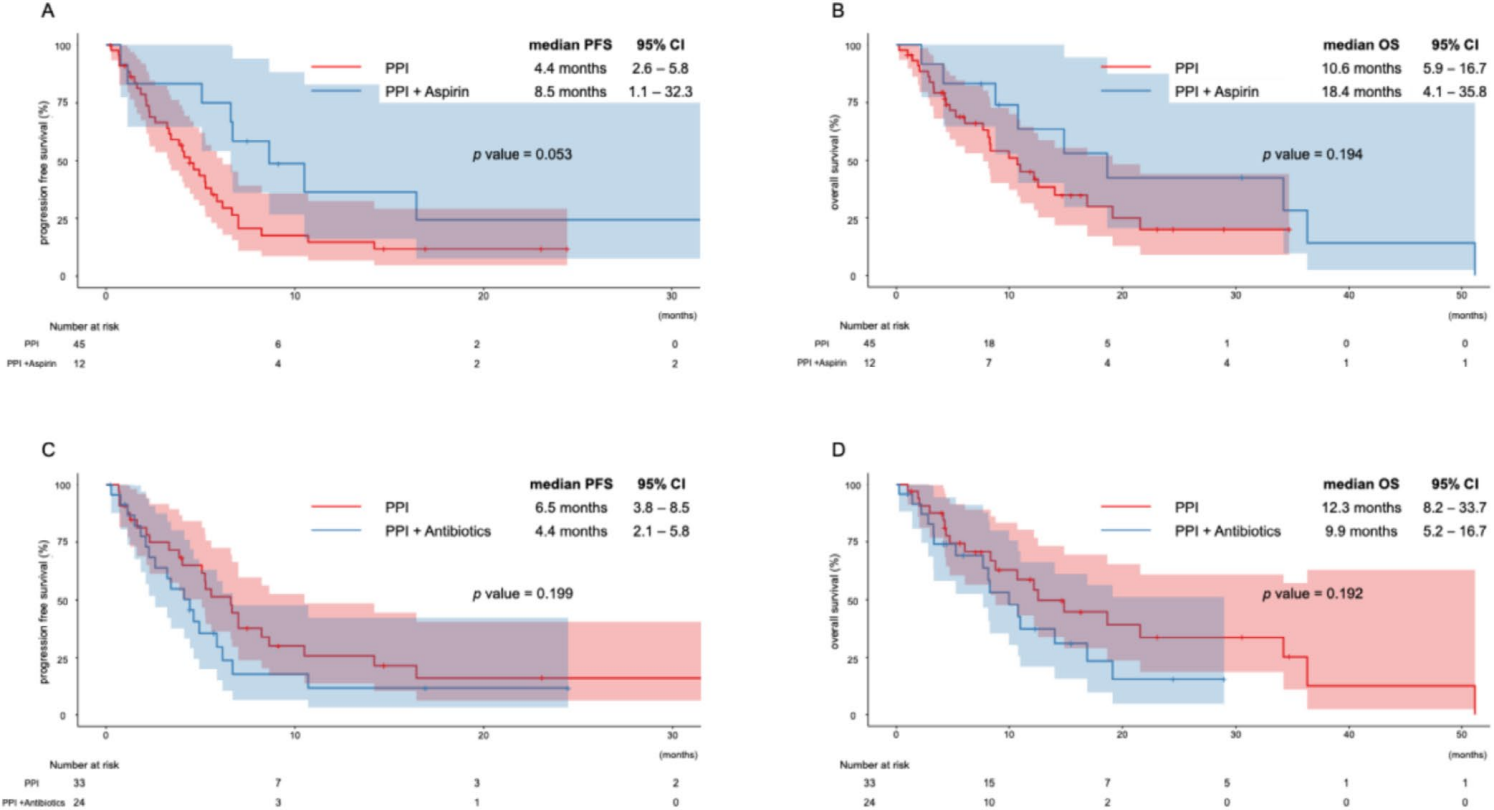
Progression‐free survival (PFS) and overall survival (OS) with and without the combination of PPIs and other drugs. (A) PFS: Comparison between the PPIs group and the PPIs + aspirin group. (B) OS: Comparison between the PPIs group and the PPIs + aspirin group. (C) PFS: Comparison between the PPIs group and the PPIs + Antibiotics group. (D) OS: Comparison between the PPIs group and the PPIs + antibiotics group.

The median PFS and OS in the PPI‐only group were 4.4 months (95% CI: 2.6–5.8) and 10.6 months (95% CI: 5.9–16.7), respectively. In contrast, patients receiving both PPI and low‐dose aspirin had a median PFS of 8.5 months (95% CI: 1.1–32.3) and median OS of 18.4 months (95% CI: 4.1–35.8). Although the differences in PFS (*p* = 0.053) and OS (*p* = 0.194) did not reach statistical significance, the PPI + aspirin group exhibited a trend toward improved outcomes (Figure 3A,B).

Conversely, patients receiving both PPI and antibiotics had a median PFS of 4.4 months (95% CI: 2.1–5.8) and OS of 9.9 months (95% CI: 5.2–18.7), compared with 6.5 months (95% CI: 3.8–8.5) and 12.3 months (95% CI: 8.2–33.7), respectively, in the PPI‐only group. Although these differences were also not statistically significant (PFS: *p* = 0.199; OS: *p* = 0.192), there was a trend toward worse outcomes in the PPI + antibiotic group (Figure 3C,D).

No consistent trends were observed in PFS or OS when PPI was combined with other concomitant drugs, such as statins, biguanides, NSAIDs, or probiotics (Figure S4).

## 4 Discussion

This retrospective study evaluated the effects of baseline use of commonly prescribed concomitant drugs on the efficacy and safety of PD‐1/PD‐L1 inhibitor‐based therapy in patients with NSCLC. We found that PPI use at treatment initiation was significantly associated with worse PFS and OS, while low‐dose aspirin use was independently associated with improved PFS. Notably, patients who received both PPI and aspirin demonstrated a non‐significant but numerically favorable outcome compared to those receiving PPI alone. In contrast, no consistent survival impact was observed for other drugs, including NSAIDs, statins, biguanides, antibiotics, or probiotics.

The negative association between PPI use and ICI efficacy observed in our study aligns with previous reports suggesting that gastric acid suppression may impair antitumor immunity by disrupting the gut microbiota [15, 16]. By raising gastric pH, PPIs can allow overgrowth of oral bacteria and reduce beneficial intestinal commensals, potentially affecting systemic immune homeostasis. Although conflicting data exist regarding the clinical impact of PPIs on ICI outcomes, our findings add to the growing body of evidence supporting the cautious use of PPIs during immunotherapy, especially in patients without a clear indication.

Conversely, low‐dose aspirin showed a favorable effect on PFS. Aspirin's antitumor activity has been attributed to its irreversible inhibition of COX‐1/2 and sustained antiplatelet effects, which may promote immune surveillance by enhancing T‐cell infiltration and reducing immune evasion, and mitigating the immunosuppressive tumor microenvironment [17, 18, 19, 20]. These mechanisms could synergize with ICIs. Importantly, as reported previously, NSAIDs—though also COX inhibitors—did not show similar benefits in our cohort, likely due to their reversible and selective block of COX‐2, which may have less impact on immune modulation [21, 22].

We also evaluated the combined impact of PPIs with other drugs. While the combination of PPI and aspirin tended to attenuate the negative impact observed with PPI alone, the combination of PPI with antibiotics was associated with a pattern suggestive of poorer outcomes. Although these effects were not statistically significant, this observation is biologically plausible given the established role of both PPIs and antibiotics in gut microbiota disruption [23, 24]. This supports the hypothesis that overlapping dysbiosis may further compromise ICI efficacy.

While many previous studies have examined the impact of individual drugs on ICIs, few have assessed the combined effects of multiple concomitant drugs. To our knowledge, this is the first study to systematically evaluate the interaction between PPIs and aspirin, as well as other drug combinations, in the context of PD‐1/PD‐L1 inhibitor therapy. Given the increasing prevalence of polypharmacy in oncology—particularly among older adults—our findings highlight the need for careful drug review at the initiation of immunotherapy.

This study has several limitations. First, it was a single‐center retrospective analysis with a limited sample size, which may restrict generalizability. Second, we evaluated only baseline drug use; data on duration, dosage, or changes during treatment were unavailable, and exposure was defined as active prescription at ICI initiation (except for antibiotics, which were assessed within 30 days prior to therapy). Due to inconsistent documentation, we were unable to perform a detailed assessment of drug exposure in terms of duration or dosage. Third, although we excluded patients receiving systemic corticosteroids for brain metastases or autoimmune disease, we did not systematically capture transient corticosteroid use related to chemotherapy premedication, which may affect immune response. Fourth, residual confounding by indication is possible, as patients prescribed certain drugs (e.g., PPIs or aspirin) may differ systematically in comorbidities or disease severity. Finally, microbiota analysis was not performed, and mechanistic insights remain speculative.

Despite these limitations, our study suggests that PPI use may impair, and low‐dose aspirin may enhance, the efficacy of PD‐1/PD‐L1 inhibitors in NSCLC. These findings underscore the clinical importance of reviewing and optimizing concomitant drugs prior to initiating immunotherapy. Future prospective studies, ideally incorporating microbiome profiling and pharmacodynamic data, are warranted to validate our findings and further elucidate the mechanisms underlying these drug–ICI interactions. In particular, integrating 16S rRNA sequencing and immune profiling could help clarify how specific drug combinations affect the host–microbiota–immune axis during immunotherapy.

## Author Contributions

**Masafumi Saiki:** conceptualization, investigation, data curation, formal analysis, writing – original draft, writing – review and editing. **Kazuho Takusagawa:** writing – review and editing. **Nozomu Takahashi:** writing – review and editing. **Kenta Homma:** writing – review and editing. **Tsukasa Satoh:** writing – review and editing. **Satoshi Furuya:** writing – review and editing. **So Shimamura:** writing – review and editing. **Chisa Omori:** writing – review and editing. **Hiroki Ohkoshi:** writing – review and editing. **Yuki Hoshino:** writing – review and editing. **Yoshinori Uchida:** writing – review and editing. **Shinnosuke Ikemura:** writing – review and editing. **Kenzo Soejima:** supervision, conceptualization, project administration, funding acquisition, writing – review and editing.

## Disclosure

The authors have nothing to report.

## Conflicts of Interest

The authors declare no conflicts of interest.

## Supporting information


**Figure S1:** Consort diagram showing the inclusion and exclusion of patients assessed for eligibility in this study.

**Figure S2:** Stratified analyses of PFS according to PD‐L1 tumor proportion score (< 1%, 1%–49%, and ≥ 50%).

**Figure S3:** Stratified analyses of OS according to PD‐L1 tumor proportion score (< 1%, 1%–49%, and ≥ 50%).

**Figure S4:** Progression‐free survival (PFS) and overall survival (OS) with and without the combination of PPIs and other drugs.

**Table S1:** Incidence of irAE.

**Table S2:** Interaction analysis between PPI use and age or PS for PFS and OS.

## Data Availability

The data that support the findings of this study are not publicly available due to privacy and ethical restrictions. Data may be available from the corresponding author upon reasonable request and with permission from the University of Yamanashi Hospital.
