# Supplementary material for: Effect of the Combination of Concomitant Drugs on Efficacy of Immune Checkpoint Inhibitors in Non‐Small Cell Lung Cancer
Source: Cancer Rep (Hoboken). 2025 Nov 6;8(11):e70399. doi: 10.1002/cnr2.70399 (PMC12590243; doi:10.1002/cnr2.70399)
Supplement: Supplementary file 1 — Figure S1: Consort diagram showing the inclusion and exclusion of patients assessed for eligibility in this study. [file CNR2-8-e70399-s006.pptx]

## Slide 1
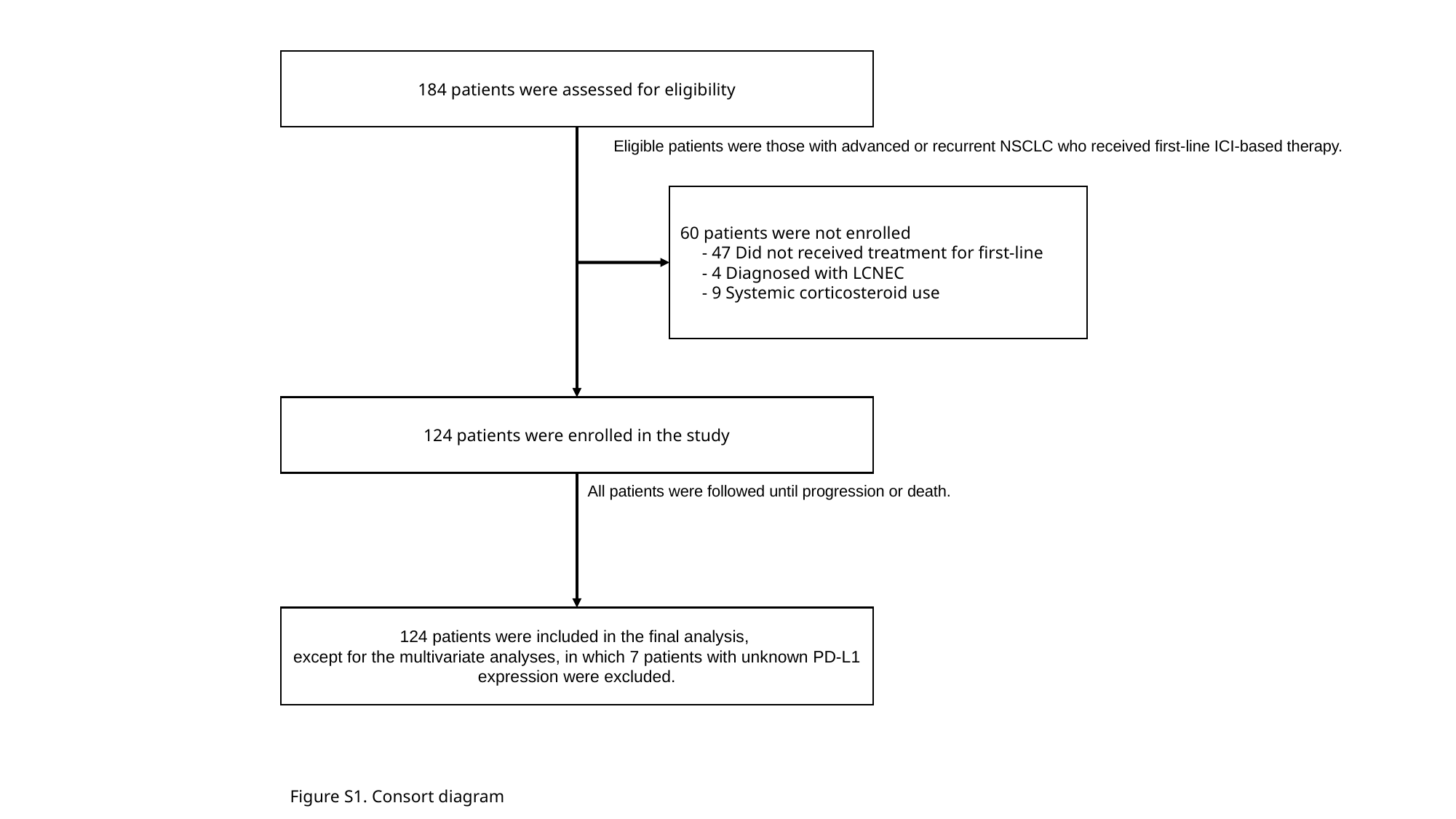

184 patients were assessed for eligibility
Eligible patients were those with advanced or recurrent NSCLC who received first-line ICI-based therapy.
60 patients were not enrolled
 - 47 Did not received treatment for first-line
 - 4 Diagnosed with LCNEC
 - 9 Systemic corticosteroid use
124 patients were enrolled in the study
All patients were followed until progression or death.
124 patients were included in the final analysis,
except for the multivariate analyses, in which 7 patients with unknown PD-L1 expression were excluded.
Figure S1. Consort diagram
