# Supplementary material for: Effect of the Combination of Concomitant Drugs on Efficacy of Immune Checkpoint Inhibitors in Non‐Small Cell Lung Cancer
Source: Cancer Rep (Hoboken). 2025 Nov 6;8(11):e70399. doi: 10.1002/cnr2.70399 (PMC12590243; doi:10.1002/cnr2.70399)
Supplement: Supplementary file 2 — Figure S2: Stratified analyses of PFS according to PD‐L1 tumor proportion score (< 1%, 1%–49%, and ≥ 50%). [file CNR2-8-e70399-s005.pptx]

## Slide 1
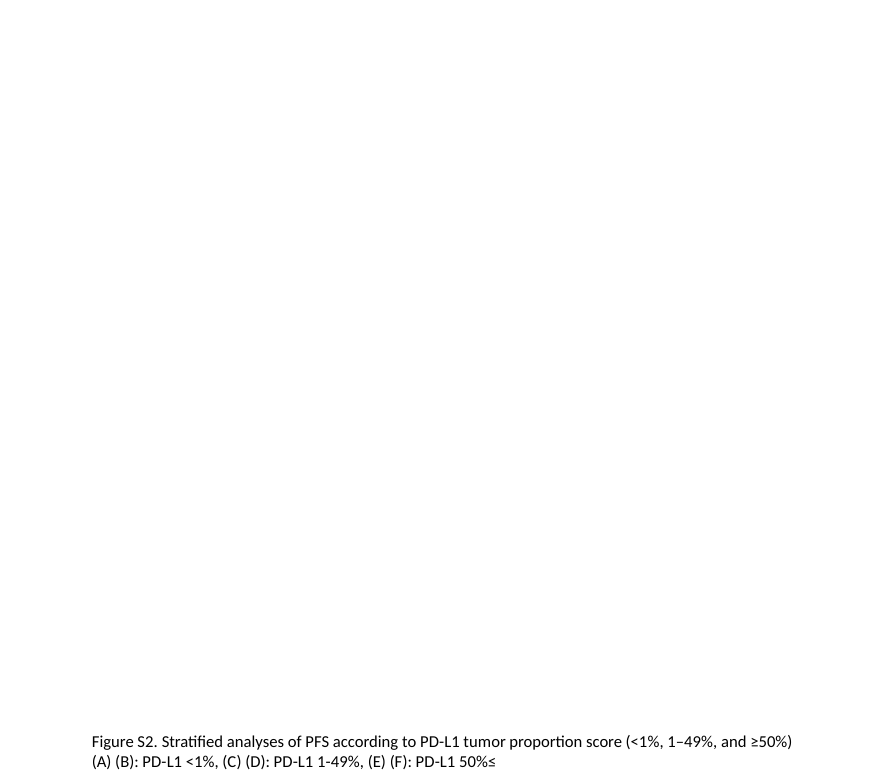

Figure S2. Stratified analyses of PFS according to PD-L1 tumor proportion score (<1%, 1–49%, and ≥50%)
(A) (B): PD-L1 <1%, (C) (D): PD-L1 1-49%, (E) (F): PD-L1 50%≤
