# Supplementary material for: Effect of the Combination of Concomitant Drugs on Efficacy of Immune Checkpoint Inhibitors in Non‐Small Cell Lung Cancer
Source: Cancer Rep (Hoboken). 2025 Nov 6;8(11):e70399. doi: 10.1002/cnr2.70399 (PMC12590243; doi:10.1002/cnr2.70399)
Supplement: Supplementary file 4 — Figure S4: Progression‐free survival (PFS) and overall survival (OS) with and without the combination of PPIs and other drugs. [file CNR2-8-e70399-s001.pptx]

## Slide 1
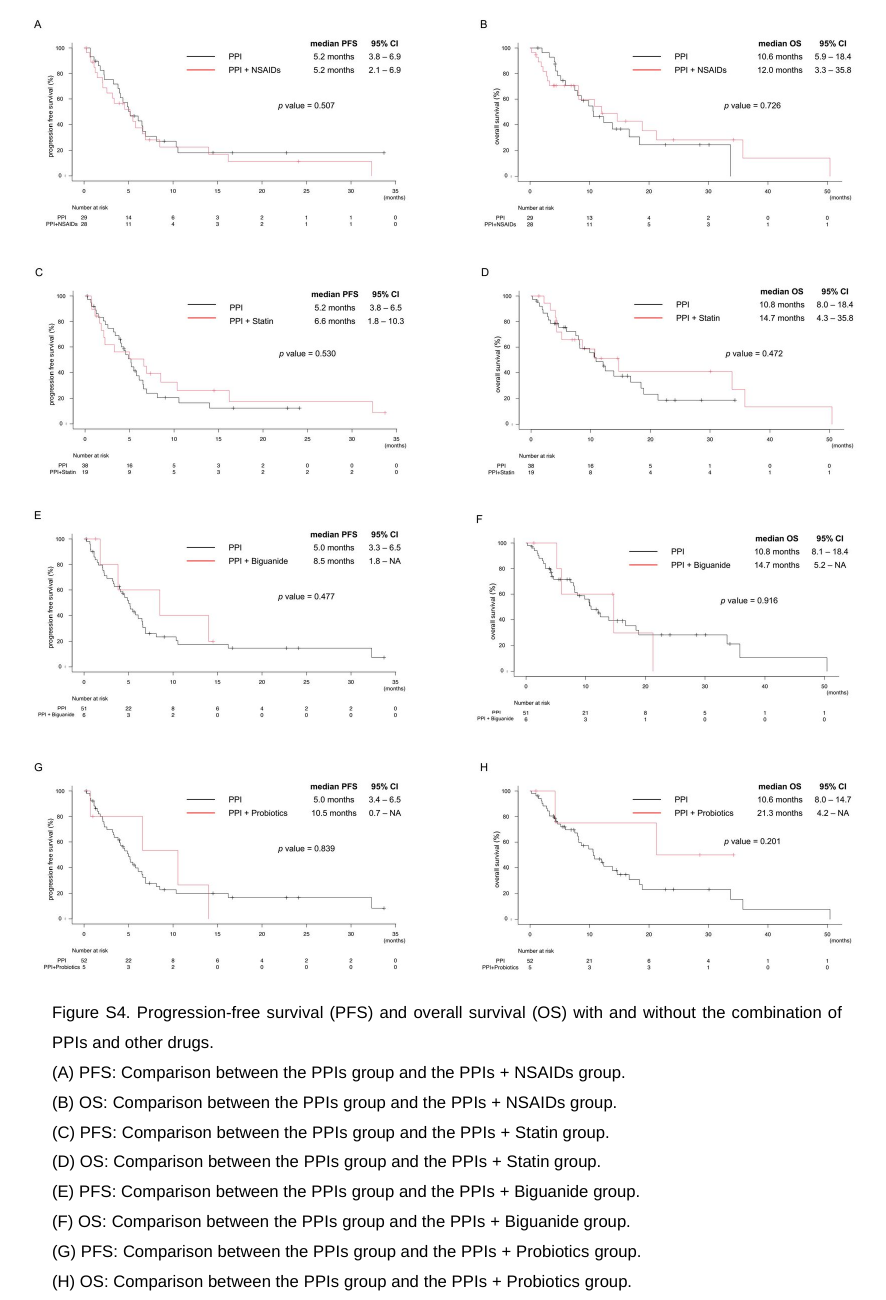

Figure S4. Progression-free survival (PFS) and overall survival (OS) with and without the combination of PPIs and other drugs.
(A) PFS: Comparison between the PPIs group and the PPIs + NSAIDs group.
(B) OS: Comparison between the PPIs group and the PPIs + NSAIDs group.
(C) PFS: Comparison between the PPIs group and the PPIs + Statin group.
(D) OS: Comparison between the PPIs group and the PPIs + Statin group.
(E) PFS: Comparison between the PPIs group and the PPIs + Biguanide group.
(F) OS: Comparison between the PPIs group and the PPIs + Biguanide group.
(G) PFS: Comparison between the PPIs group and the PPIs + Probiotics group.
(H) OS: Comparison between the PPIs group and the PPIs + Probiotics group.
