# Supplementary material for: Effect of the Combination of Concomitant Drugs on Efficacy of Immune Checkpoint Inhibitors in Non‐Small Cell Lung Cancer
Source: Cancer Rep (Hoboken). 2025 Nov 6;8(11):e70399. doi: 10.1002/cnr2.70399 (PMC12590243; doi:10.1002/cnr2.70399)
Supplement: Supplementary file 5 — Table S1: Incidence of irAE. [file CNR2-8-e70399-s002.docx]

Table S1. Incidence of irAE

| irAE | any grade | grade 3≤ |
| --- | --- | --- |
| Any events | 84 | 26 |
| Pneumonitis | 33 | 13 |
| Rush | 14 | 1 |
| Adrenal insufficiency | 7 | 1 |
| Hypothyroidism | 7 | 0 |
| Colitis | 6 | 3 |
| Liver dysfunction | 4 | 1 |
| Arthritis | 3 | 1 |
| CRS | 2 | 2 |
| Infusion reaction | 2 | 1 |
| Pericarditis | 1 | 0 |
| Renal dysfunction | 1 | 1 |
| Type 1 DM | 1 | 1 |
| Other | 3 | 1 |

Abbreviation; AE: adverse event; CRS: cytokine released syndrome; DM: diabetes mellitus.
