# Supplementary material for: Effect of the Combination of Concomitant Drugs on Efficacy of Immune Checkpoint Inhibitors in Non‐Small Cell Lung Cancer
Source: Cancer Rep (Hoboken). 2025 Nov 6;8(11):e70399. doi: 10.1002/cnr2.70399 (PMC12590243; doi:10.1002/cnr2.70399)
Supplement: Supplementary file 6 — Table S2: Interaction analysis between PPI use and age or PS for PFS and OS. [file CNR2-8-e70399-s004.docx]

Table S2. Interaction analysis between PPI use and age or PS for PFS and OS

|  | PFS |  |  | OS |  |
| --- | --- | --- | --- | --- | --- |
|  | HR (95%CI) | p value |  | HR (95%CI) | p value |
| PPI × Age (≥75 vs. <75) | 0.77 (0.30–1.96) | 0.58 |  | 0.60 (0.23–1.54) | 0.28 |
| PPI × PS (2–4 vs. 0–1) | 2.35 (0.78–7.08) | 0.13 |  | 2.36 (0.81–6.90) | 0.12 |

Abbreviations:

PPI, proton pump inhibitor; PS, performance status; PFS, progression-free survival; OS, overall survival; HR, hazard ratio; CI, confidence interval.
